# Causality and preventability assessment of adverse drug reactions and adverse drug events of antibiotics among hospitalized patients: A multicenter, cross-sectional study in Lahore, Pakistan

**DOI:** 10.1371/journal.pone.0199456

**Published:** 2018-06-27

**Authors:** Sadia Iftikhar, Muhammad Rehan Sarwar, Anum Saqib, Muhammad Sarfraz

**Affiliations:** 1 Akhtar Saeed College of Pharmaceutical Sciences, Lahore, Pakistan; 2 Department of Pharmacy, The Islamia University of Bahawalpur Bahawalpur, Punjab, Pakistan; 3 College of Pharmacy, Al Ain University of Science and Technology, Al Ain, Abu Dhabi, UAE; Aligarh Muslim University, INDIA

## Abstract

**Background and objectives:**

Adverse drug events (ADEs) are the fifth leading cause of death and thus responsible for a large number of hospital admissions in all over the globe. This study was aimed to assess the antibiotics associated preventability of ADEs and causality of adverse drug reactions (ADRs) among hospitalized patients.

**Methods:**

A prospective, cross-sectional, observational study was conducted in four tertiary care public sector hospitals of Lahore, Pakistan. Study population consisted of hospitalized patients who were prescribed with one or more antibiotics. Data were collected between 1^st^ January, 2017 and 31^st^ June, 2017 from 1,249 patients (384 patients aged ≤ 18 years and 865 patients aged >18 years). Schumock and Thornton scale was used to assess the preventability of the ADEs. Medication errors (MEs) that caused preventable ADEs were assessed by MEs tracking form while Naranjo score was used to evaluate the causal relation of ADRs with the antibiotics. Statistical Package for the Social Sciences (IBM SPSS Statistics for Windows, Version 21.0. Armonk, NY: IBM Corp.) and Microsoft Excel (MS Office, 2010) were used for data analysis.

**Results:**

2,686 antibiotics were prescribed to 1,249 patients. Among them, fluoroquinolones (11.8%), macrolides (11.6%) and cephalosporins (10.9%) were the most frequently prescribed antibiotics. The most affected organ system by antibiotics associated ADEs was gastrointestinal tract. A total of 486 ADEs were found. The preventability assessment revealed that most of the ADEs (58.4%) were preventable (43.6% of the ADEs were definitely preventable while 14.8% were probably preventable) and caused by MEs including wrong drug (40.1%) and monitoring errors (25.0%), during the stage of physician ordering (22.2%) and patient monitoring (21.1%). The errors were caused due to non-adherence of policies (38.4%) and lack of information about antibiotics (32%). Most of the non-preventable ADEs or ADRs among adults and children were “probable” (35.5%) and “possible” (35.8%), respectively. Logistic regression analysis revealed that ADEs were significantly less among females (OR = 0.047, 95%CI = 0.018–0.121, *p-value* = <0.001), patients aged 18–52 years (OR = 0.041, 95%CI = 0.013–0.130, *p-value* = <0.001), tuberculosis patients (OR = 0.304, 95%CI = 0.186–0.497, *p-value* = <0.001), patients with acute respiratory tract infections (OR = 0.004, 95%CI = 0.01−0.019, *p-value* = <0.001) and among the patients prescribed with 2 antibiotics per prescription (OR = 0.455, 95%CI = 0.319–0.650, *p-value* = <0.001).

**Conclusion:**

According to preventability assessment most of the ADEs were definitely preventable and caused by MEs due to non-adherence of policies and lack of information about antibiotics. The causality assessment of non-preventable ADEs showed that most of the ADRs were probable and possible.

## Introduction

According to the World Health Organization (WHO), adverse drug reaction (ADR) is defined as “any response to a drug which is noxious, unintended, and may occur at doses normally used in man for the prophylaxis, diagnosis, or therapy of disease [1].” According to the National Coordinating Council for Medication Error Reporting and Prevention (NCC MERP), adverse drug events (ADEs) are the injuries that are either related to the dose of the drugs or the medical interventions [2]. Both these definitions have subtle differences; however these are crucial in making decisions during routine clinical practices. ADE is an adverse outcome that occurs after the use of a drug, but which may or may not be linked to use of the drug. Therefore, all ADRs are referred to as ADEs, but that not all ADEs can be considered as ADRs. Medication errors (MEs) are those that occur at any stage during the processing of medication [3]. The medication associated ADRs are mostly responsible for the higher rate of morbidity and mortality. Also, ADRs are ranked as the seventh recurrent cause of mortality because one out of every seventh inpatient experiences ADRs during his stay at the hospital [4]. Findings of a meta-analysis showed that annually 1,00,000 patients die in the USA due to ADRs [5]. According to an estimate of a Swedish study, 3.1% of deaths in the general population (encompassing subjects who died in and outside the hospitals) are due to ADRs [6]. The causality assessment is used to establish a probable relationship between medication and ADRs [7]. The scientific term that encompasses the method of comprehension, recognition and prevention of ADEs is known as pharmacovigilance [8]. The basic purpose of pharmacovigilance is to ensure patient safety by preventing these untoward effects [9]. The identification of ADEs still remains a major challenge for physicians [7]. Similar to developing countries, the prevalence of ADEs is common in developed regions. A study revealed the prevalence rate of ADEs among hospitalized patients of England as 3.2%, Germany as 4.8% and the United States of America (USA) as 5.6% [10]. Adverse drug events (ADEs) are considered as the fifth leading cause of death globally, and their poor monitoring and reporting system has worsen the situation [11]. In developing countries like Pakistan, the antibiotics are frequently used in the inpatient departments of the healthcare settings. Furthermore, due to physiological and pharmacokinetic variations, antibiotic associated ADEs are common in pediatric and geriatric patients [12]. According to an estimate, almost 50% of the inpatients receive one antibiotic agent during their hospital stay and in 20–30% cases, the use of antibiotics is not therapeutically necessary [13,14]. Most of the ADEs are preventable because they occur as a result of medication errors at any stage of medication processing i.e., prescribing, transcribing, dispensing, administering, adherence, or monitoring a drug [15]. A study conducted in Netherlands reported that ADEs pertaining to medication interventions account for 0.2% per hospital admission [16]. The misuse of antibiotics is a matter of concern because of its association with a number of potentially harmful ADEs which may include end-organ damage, hypersensitivity reactions, and super-infections with various antibiotic-resistant organisms [17–19].

In Pakistan, both public and private sector hospitals lack proper pharmacovigilance system and there is unavailability of data for antibiotic-associated ADEs among hospitalized patients on regional, zonal, and national level. Previously published studies round the globe also lack such estimates. A study conducted by Shehab, et al. reported that 19% patients visited emergency department due to antibiotics-associated ADEs [20]. Inpatients are more likely to experience ADEs due to various inevitable reasons which may include 1) the frequent administration of multiple antibiotics to the hospitalized patients [21] and 2) mostly, the inpatients comprises of pediatrics, geriatrics or patients having various co-morbidities and all these patients have high risk of developing ADEs [22,23].

The detection of antibiotic associated ADEs are essential for patient safety. Although numerous studies have been conducted in other countries except Pakistan to determine the preventability associated with ADEs and causal relation of ADRs with antibiotics but those were either limited to a single class of antibiotics or single infection of bacterial origin [24–27]. Therefore, the present study aims to determine the preventability and the reasons of ADEs along with the causal relationship of ADRs with antibiotics that are commonly administered to the hospitalized patients in Pakistan.

## Methods

### Study design and settings

A prospective, cross-sectional, observational study was conducted in four public tertiary care hospitals (Mayo hospital, Jinnah hospital, General hospital, and Services hospital) of Lahore, Punjab province of Pakistan. All the study settings lack pharmacovigilance centers and ADEs registers. The detail description about study settings have been mentioned in S1 Appendix.

### Study population and sample size

According to latest Pakistani census, the total population living in Pakistan is 201,995,540 [28]. Lahore is the most populous city of Punjab province of Pakistan, with a total population of 11,126, 285 [29]. The study population included the patients of all age groups, admitted in general internal medicine ward and pediatric ward, prescribed with antibiotics on the basis of differential diagnosis for ≥24 hours. All those patients were excluded who had medical history of cardiac diseases, hepatic and renal insufficiencies, ear, nose and throat (ENT) disorders. According to hospitals records, a total of 14,592 patients were admitted in internal medicine and pediatric departments during the 6 months of study period. Among them, 1,249 patients (age range 6 to 52 years) met the inclusion criteria of this study.

### Data collection

A data collection form [S2 Appendix] was developed which consisted of seven parts: 1) characteristics of the patients, 2) diagnosis, 3) recommended antibiotics, 4) medication errors, 5) causality assessment by Naranjo score, 6) preventability assessment and 7) the effect of ADRs on organ system (if any). The Anatomical Therapeutic Chemical (ATC) classification system [30] was used for the coding of antibiotics. SPSS version 21.0 was used for calculation of reliability coefficients. Internal consistency was measured by Cronbach’s alpha, while reproducibility was evaluated by using intra-class correlation for each item in the scales, with acceptable values ≥0.6. Calculation for Cronbach’s alpha was set at 0.76 for Schmuck and Thornton scale, 0.74 for ME tracking form, and 0.78 for Naranjo score. A pilot study was undertaken between November and December 2016 for pre-testing the study instrument. Data were collected between 1st January, 2017 and 31st June, 2017 according to the objectives of the study. The investigational team included a medical practitioner, pharmacist and a nurse. A total of 8 investigational teams were made. As there was no heterogeneity in the training of all team members, so they were consistent in the determination of ADEs and ADRs. Two investigational teams were assigned to each hospital; one for internal medicine ward and other for pediatric ward.

The review of medical records was conducted on daily basis until the patients were discharged from the respective ward. This enables the investigators to scrutinize data from pertinent lab reports, physician’s progress notes, patient’s medication records (dose, dosage form, frequency and duration of prescribed antibiotics), physician’s order, multidisciplinary progress notes and discharge summaries. All the sign and symptoms that appeared after the use of antibiotics were also recorded. The teams also participated in ward rounds and checked the presence of any alerts for MEs and ADEs. As the attending physicians and clinical pharmacists were having expertise in the field of pharmacovigilance and antibiotic surveillance, so their opinions were also taken in account before reaching the final decision about the occurrence of ADEs.

Note: In this study ADEs refer to injuries which are either caused by the drug (i.e., ADRs or non-preventable ADEs) or by the use of the therapeutic agents (i.e., medication errors or preventable ADEs) while ADRs refer to the definition given by Edwards and Aronson i.e., unpleasant or harmful reactions that have causal relation with the medicinal product, predict untoward outcomes from future administration and demands withdrawal from therapy, alteration of dosage regimen and specific treatments [31]. British National Formulary was used for confirming the ADRs [32]. MEs are those that occur during the processing of medication i.e., prescribing, transcribing, dispensing, administering, adherence, or monitoring a drug [3]. MEs were identified by using the standard guidelines of Current Medical Diagnosis AND Treatment (CMDT) [33], National Institute of Health and Clinical Excellence (NICE) guidelines [34], British National Formulary (BNF) for children [35] and Infectious Diseases Society of Pakistan (IDSP) guidelines for antibiotic use [36]. Some other definitions of important terminologies have been mentioned in S3 Appendix.

### Outcome variables

The outcome variables included causality assessment and preventability assessment. The cases in which ADEs appeared were further analyzed for assessing the preventability by Schumock and Thornton Scale. Medication errors were found by using medication error tracking form among definitely preventable and probably preventable ADEs. Naranjo scale was used for determining the causal relationship between non-preventable ADEs and antibiotics.

#### Schumock and Thornton scale

The Schumock and Thornton criteria [37] was established for assessing the preventability of ADRs. The modified form of this criterion has been used in various studies [38–40]. It has three sections namely definitely preventable, probably preventable and non-preventable. Section A comprises of five questions while section B has four questions. All the answers are categorized as “Yes” or “No”. ADRs were “definitely preventable” if answer was “yes” to one or more questions in section A. If answers were all negative then we proceeded to section B. ADRs were “probably preventable” if answer was “yes” to one or more questions in section B. If answers were all negative then we proceeded to section C. In Section C the ADRs were non-preventable.

#### Naranjo scale

The Naranjo Scale was developed by Naranjo and coworkers from the University of Toronto [41] for assessing the likelihood of whether an ADR is due to some particular drug or other factors. This validated tool has been used in multiple studies [42–45]. This scale comprises of 10 questions that are answered “Yes”, “No”, or “Do not know”. Different point values (-1, 0, +1 or +2) are assigned to each answer. Total scores range from -4 to +13; the reaction is considered definite if the score is 9 or higher, probable if 5 to 8, possible if 1 to 4, and doubtful if 0 or less.

#### Medication error tracking form

This tool was prepared for addressing MEs in hospitals for the California Health Care Foundation Data [46]. It consisted of three sections: 1) patient information, 2) medication order information and 3) medication error categorization. The third section comprised of “medication class”, “categories” and “possible causes” of MEs. It also classified MEs into five categories: A) prescribing, B) transcribing, C) dispensing, D) administering and E) monitoring.

### Data analysis

Statistical Package for Social Sciences (IBM Corp. Released 2012. IBM SPSS Statistics for Windows Version 21.0. Armonk, NY: IBM Corp.) and Microsoft Excel (MS Office 2010) were used for data analysis. Descriptive statistics such as frequencies and percentages were used to present the data. Furthermore, logistic regression analysis was performed to figure out the factors associated with ADEs. Results were expressed as Odds Ratio (OR) accompanied by 95% Confidence Intervals (95% CI) and a p-value <0.05 was used for statistical significance of differences.

### Ethics approval and consent to participate

The ethical approval was obtained from the Pharmacy Research Ethics Committee (PREC) at Akhtar Saeed College of Pharmaceutical Sciences (Reference: 3-2016/PREC, December 22, 2016). Before conducting the study, permission was granted from the hospital administrators. The purpose and protocols of this study were thoroughly explained to every participant and their verbal consents were obtained. Written consent was not possible for most of the respondents because either they were illiterate or they had problems in reading and/or signing the consent document.

## Results

### Characteristics of the patients

A total of 1,249 patients were investigated. Among them, 57.3% were male and 69.3% were aged >18 years. 37% patients (n = 462) were prescribed antibiotics for urinary tract infections, 29% (n = 362) for acute respiratory tract infections, and 34% (n = 425) for skin and soft tissue infections (Table 1).

**Table 1:** Characteristics of patients.

Characteristics		Total patients n (%)*
Gender	Male	716 (57.3)
	Female	533 (42.7)
Age	Adults (>18 years)	865 (69.3)
	Children (≤18 years)	384 (30.7)
Co-morbidities	Diabetes	526 (42.1)
	Asthma	424 (33.9)
	Tuberculosis	137 (11.0)
	Cystic fibrosis	162 (13.0)
Antibiotic indications	Acute respiratory tract infections	362 (29.0)
	Urinary tract infections	462 (37.0)
	Skin and soft tissue infections	425 (34.0)
Number of antibiotics prescribed per prescription	1	229 (18.3)
	2	603 (48.3)
	3	417 (33.4)

*Percentages have been calculated with respect to the total sample size (n = 1249)

### Prescribing pattern of antibiotics

A total of 2,686 antibiotics were prescribed among 1,249 patients. Among them, fluoroquinolones (11.8%, n = 316), macrolides (11.6%, n = 311) and cephalosporins (10.9%, n = 292) were the most frequently prescribed antibiotics (Table 2).

**Table 2:** Antibiotics prescribed among study population.

Antibiotics Class	ATC* code	Number of patients received antibiotics (n = 1,249)	Number of prescribed antibiotics (n = 2,686)
Penicillins	J01C	194 (15.5)	261 (9.7)
Macrolides	J01FA	252 (20.2)	311 (11.6)
Cephalosporins	J01D	223 (17.9)	292 (10.9)
Fluoroquinolones	J01M	291 (23.3)	316 (11.8)
Aminoglycosides	J01G	192 (15.4)	226 (8.4)
Tetracyclines	J01AA	193 (15.5)	221 (8.2)
Lincosamide	J01FF	127 (10.2)	209 (7.8)
Carbapenem	J01DH	106 (8.5)	234 (8.7)
Glycopeptide	J01XA	91 (7.3)	214 (7.9)
Oxazolidones	J01XX	102 (8.2)	186 (6.9)
Imidazole derivatives	G01AF	113 (9.5)	216 (8.0)

* ATC = Anatomical Therapeutic Chemical Classification System

### Organ system affected by ADEs

Antibiotic associated ADEs were found in 38.9% (n = 486) patients. Overall, the most affected organ system was GIT (adults = 30.1%, children = 38%) as shown in Table 3.

**Table 3:** Effect of antibiotics on organ systems of study participants.

Antibiotics	Total ADEs N (%)	Age groups N (%)	Cardiac* N (%)	GIT^†^ N (%)	Ototoxicity^‡^ N (%)	Hematology^§^ N (%)	Hepatobiliary^||^ N (%)	Renal^¶^ N (%)	Neurotoxicity** N (%)	Others^††^ N (%)
Penicillins	62 (35.6)	Adults 38 (61.3)	0 (0.0)	19 (50.0)	0 (0.0)	2 (5.3)	5 (13.2)	2 (5.3)	4 (10.5)	6 (15.8)
		Children 24 (38.7)	0 (0.0)	15 (62.5)	0 (0.0)	0 (0.0)	0 (0.0)	0 (0.0)	6 (25.0)	3 (12.0)
Aminoglycosides	37 (26.1)	Adults 26 (70.3)	0 (0.0)	2 (7.7)	6 (23.1)	1 (3.8)	0 (0.0)	9 (34.6)	3 (11.5)	5 (19.2)
		Children 11 (29.7)	0 (0.0)	3 (27.3)	5 (45.5)	1 (9.1)	0 (0.0)	2 (18.2)	0 (0.0)	0 (0.0)
Macrolides	61 (59.8)	Adults 33 (54.1)	6 (18.2)	8 (24.2)	7 (21.2)	3 (9.1)	4 (12.1)	0 (0.0)	0 (0.0)	5 (15.2)
		Children 28 (45.9)	2 (7.1)	10 (35.7)	4 (14.3)	0 (0.0)	5 (17.9)	0 (0.0)	0 (0.0)	7 (25.0)
Cephalosporins	66 (33.7)	Adults 41 (62.1)	0 (0.0)	12 (29.3)	0 (0.0)	17 (41.5)	6 (14.6)	3 (7.3)	2 (4.9)	1 (2.4)
		Children 25 (37.9)	0 (0.0)	1 (4.0)	0 (0.0)	7 (28.0)	13 (52.0)	3 (12.0)	1 (4.0)	0 (0.0)
Fluoroquinolones	61 (67.0)	Adults 39 (63.9)	4 (10.2)	1 (2.6)	9 (23.1)	5 (12.8)	14 (35.9)	2 (5.1)	3 (7.7)	1 (2.6)
		Children 22 (36.1)	3 (13.6)	7 (31.8)	0 (0.0)	0 (0.0)	1 (4.6)	4 (18.2)	5 (22.7)	2 (9.1)
Tetracyclines	36 (34.9)	Adults 23 (63.9)	0 (0.0)	9 (39.1)	0 (0.0)	0 (0.0)	0 (0.0)	0 (0.0)	6 (26.1)	8 (34.8)
		Children 13 (36.1)	0 (0.0)	10 (76.9)	0 (0.0)	0 (0.0)	0 (0.0)	0 (0.0)	0 (0.0)	3 (23.1)
Lincosamide	26 (26.8)	Adults 17 (65.4)	0 (0.0)	13 (76.5)	0 (0.0)	4 (23.5)	0 (0.0)	0 (0.0)	0 (0.0)	0 (0.0)
		Children 9 (34.6)	0 (0.0)	6 (66.7)	0 (0.0)	0 (0.0)	0 (0.0)	0 (0.0)	0 (0.0)	3 (33.3)
Carbapenem	34 (36.9)	Adults 23 (67.7)	0 (0.0)	11 (47.8)	0 (0.0)	8 (34.8)	4 (17.4)	0 (0.0)	0 (0.0)	0 (0.0)
		Children 11 (32.3)	0 (0.0)	5 (45.5)	0 (0.0)	2 (18.2)	4 (36.4)	0 (0.0)	0 (0.0)	0 (0.0)
Glycopeptide	37 (44.1)	Adults 19 (51.4)	0 (0.0)	4 (21.1)	0 (0.0)	0 (0.0)	0 (0.0)	9 (47.4)	0 (0.0)	6 (31.6)
		Children 18 (48.6)	0 (0.0)	0 (0.0)	8 (44.4)	7 (38.9)	0 (0.0)	1 (5.6)	0 (0.0)	2 (11.1)
Oxazolidones	29 (32.2)	Adults 21 (72.4)	0 (0.0)	5 (23.8)	0 (0.0)	0 (0.0)	0 (0.0)	0 (0.0)	9 (42.9)	7 (33.3)
		Children 8 (27.6)	0 (0.0)	4 (50.0)	0 (0.0)	1 (12.5)	0 (0.0)	0 (0.0)	0 (0.0)	3 (37.5)
Imidazole derivative	37 (47.4)	Adult 22 (59.5)	0 (0.0)	7 (31.8)	0 (0.0)	0 (0.0)	0 (0.0)	0 (0.0)	13 (59.1)	2 (9.1)
		Children 15 (40.5)	0 (0.0)	9 (60.0)	0 (0.0)	0 (0.0)	0 (0.0)	0 (0.0)	0 (0.0)	6 (40.0)
Total	486 (38.9)	Adults 302 (62.1)	10 (3.3)	91 (30.1)	22 (7.3)	40 (13.3)	33 (10.9)	25 (8.3)	40 (13.2)	41 (13.6)
		Children 184 (37.9)	5 (2.7)	70 (38.0)	17 (9.2)	18 (9.8)	23 (12.5)	10 (5.4)	12 (6.5)	29 (15.8)

*QTc >440 millisecond (ms) in males or >460 ms in females in the absence of preexisting arrhythmias, based on ≥2 electrocardiograms;

^†^Abdominal discomfort, nausea and vomiting associated with antibiotic administration, in the absence of an alternate explanation;

^‡^the ability of speech discrimination was diminished upon administration of antibiotics;

^§^Developed in the absence of myelosuppressive drugs and characterized as thrombocytopenia (decrease in platelet count < 150 × 103/μL), anemia (decrease in hemoglobin level <10 g/dL) and leukopenia (decrease in white blood cells level < 4500 cells/ μL);

^||^Characterized as increase in total bilirubin (>3 mg/dL) or alanine transaminase (>3 times patient’s baseline) or aspartate transaminase (>3 times patient’s baseline) when there was no preexisting hepatobiliary disease;

^¶^Characterized as high level of serum creatinine i.e. > 1.5 time baseline when there was no preexisting acute kidney injury (e.g. sepsis) or exposure to nephrotoxic drug or intravenous contrast;

**Demonstrated as antibiotic associated toxicity, peripheral neuropathy, seizures (when there was no preexisting neurologic condition) or altered mental condition;

^††^Other ADRs among children may include penicillins-associated hypersensitivity; macrolides-associated rashes and Stevens-Johnson syndrome; Fluoroquinolones-associated arthralgia and tendon disorders; tetracyclines-associated tooth discoloration and enamel defects; Lincosamide-associated metallic taste; Glycopeptide-associated flushing and maculopapular rash; Oxazolidones-associated red man syndrome, pruritus and oral candidiasis; imidazole-associated taste disturbance. Other ADRs among adults may include penicillins-associated hypersensitivity; aminoglycosides-associated stomatitis; macrolides-associated pancreatitis; cephalosporins-associated Stevens-Johnson syndrome, pruritus and urticaria; Fluoroquinolones-associated hypotension; Tetracyclines-associated rash, dermatitis and angioedema; Glycopeptide-associated red man syndrome and phlebitis; Oxazolidones-associated taste disturbance and polyuria; imidazole-associated taste disturbance and neuropathy.

### Preventability assessment

More than half (n = 284, 58.4%) of the ADEs were preventable (43.6% of the ADEs were definitely preventable while 14.8% were probably preventable) and less than half (n = 202, 41.6%) were non-preventable ADEs or ADRs according to modified Schumock and Thornton criteria (Table 4).

**Table 4:** Preventability assessment.

Schumock and Thornton criteria	Adult (n = 302)	Child (n = 184)	Total (n = 486)
**Section A: Definitely preventable ADEs**			
Was there a history of allergy or previous reaction to the drug?	6 (1.9)	5 (2.7)	11 (2.3)
Was the drug involved inappropriate for the patient’s clinical condition?	49 (16.2)	26 (14.1)	75 (15.4)
Was the dose, route, or frequency of administration inappropriate for patient’s age, weight or disease state?	39 (12.9)	23 (12.5)	62 (12.8)
Was toxic serum drug concentration or lab monitoring test documented?	27 (8.9)	13 (7.1)	40 (8.2)
Was there a known treatment for ADEs?	13 (4.3)	11 (5.9)	24 (4.9)
***Total***	***134 (44*.*4)***	***78 (42*.*4)***	***212 (43*.*6)***
**Section B: Probably preventable ADEs**			
Was therapeutic drug monitoring or other necessary lab test not performed?	28 (9.3)	4 (2.2)	32 (6.6)
Was the drug interaction involved in ADEs?	7 (2.3)	5 (2.7)	12 (2.5)
Was poor compliance involved in ADE?	8 (2.7)	9 (4.9)	17 (3.5)
Were preventative measures not prescribed or administered to the patient?	4 (1.3)	7 (3.8)	11(2.3)
***Total***	***47 (15*.*6)***	***25 (13*.*6)***	***72 (14*.*8)***
***Total (preventable ADEs)***	***181 (59*.*9)***	***103 (55*.*9)***	***284 (58*.*4)***
**Section C: Non-preventable ADEs or ADRs**			
If all the above criteria not fulfilled.	121 (40.1)	81 (44.0)	202 (41.6)

Among preventable ADEs (58.4%, n = 284), most of the definitely preventable ADEs were found in those patients who received macrolides and cephalosporins, while probably preventable ADEs were found among those who were administered fluoroquinolones and lincosamide. Non-preventable ADEs (41.6%, n = 202) were found among those patients who were prescribed with cephalosporins and fluoroquinolones (Table 5).

**Table 5:** ADEs with respect to class of prescribed antibiotics.

Antibiotics	ATC code	Definitely preventable ADEs			Probably preventable ADEs			Non-preventable ADEs		
		Adults (n = 134)	Child (n = 78)	Total (n = 212)	Adults (n = 47)	Child (n = 25)	Total (n = 72)	Adult (n = 121)	Child (n = 81)	Total (n = 202)
Penicillins	J01C	15 (11.2)	10 (12.8)	25 (11.8)	6 (12.8)	3 (12.0)	9 (12.5)	17 (14.1)	11 (13.6)	28 (13.9)
Macrolides	J01FA	17 (12.7)	14 (17.9)	31 (14.6)	3 (6.4)	1 (4.0)	4 (5.6)	13 (10.7)	13 (16.1)	26 (12.9)
Cephalosporins	J01D	20 (14.9)	11 (14.1)	31 (14.6)	2 (4.3)	4 (16.0)	6 (8.3)	19 (15.7)	10 (12.4)	29 (14.4)
Fluoroquinolones	J01M	10 (7.5)	11 (14.1)	21 (9.9)	11 (23.4)	2 (8.0)	13 (18.1)	18 (14.9)	9 (11.1)	27 (13.4)
Aminoglycosides	J01G	12 (8.9)	4 (5.1)	16 (7.5)	4 (8.5)	2 (8.0)	6 (8.3)	10 (8.3)	5 (6.2)	15 (7.4)
Tetracyclines	J01AA	7 (5.2)	6 (7.7)	13 (6.1)	7 (14.9)	1 (4.0)	8 (11.1)	9 (7.4)	6 (7.4)	15 (7.4)
Lincosamide	J01FF	4 (2.9)	1 (1.3)	5 (2.4)	6 (12.8)	4 (16.0)	10 (13.9)	7 (5.8)	4 (4.9)	11 (5.5)
Carbapenem	J01DH	13 (9.7)	5 (6.4)	18 (8.5)	1 (2.1)	1 (4.0)	2 (2.8)	9 (7.4)	5 (6.2)	14 (6.9)
Glycopeptide	J01XA	9 (6.7)	8 (10.3)	17 (8.0)	2 (4.3)	2 (8.0)	4 (5.6)	8 (6.6)	8 (9.9)	16 (7.9)
Oxazolidones	J01XX	12 (8.9)	4 (5.1)	16 (7.5)	3 (6.4)	1 (4.0)	4 (5.6)	6 (4.9)	3 (3.7)	9 (4.5)
Imidazole derivatives	G01AF	15 (11.2)	4 (5.1)	19 (8.9)	2 (4.3)	4 (16.0)	6 (8.3)	5 (4.1)	7 (8.6)	12 (5.9)

### Medication errors

In all the preventable cases of ADEs (n = 284), the wrong drug errors (n = 114, 40.1%) and monitoring errors (n = 71, 25%) were more commonly found among the study population. Physician ordering (22.2%, n = 63) and patient monitoring (21.1%, n = 60) were the most common stages of medication errors. These errors were mainly caused due to non-adherence of policies and procedures (38.4%, n = 109) and lack of information about antibiotics (32%, n = 91) (Table 6).

**Table 6:** Antibiotic associated errors in study population.

Variables		Adult (n = 181)	Child (n = 103)	Total (n = 284)
**Type of medication errors**	Wrong drug	71 (39.2)	43 (41.7)	114 (40.1)
	Wrong dose	24 (13.3)	17 (16.5)	41 (14.4)
	Wrong route	4 (2.2)	1 (0.9)	5 (1.8)
	Wrong time	11 (6.1)	4 (3.9)	15 (5.3)
	Deteriorated drug	1 (0.6)	2 (1.9)	3 (1.1)
	Omission	9 (4.9)	6 (5.8)	15 (5.3)
	Wrong dosage form	1 (0.6)	2 (1.9)	3 (1.1)
	Non-adherence	8 (4.4)	9 (8.7)	17 (5.9)
	Monitoring error	52 (28.7)	19 (18.5)	71 (25.0)
**Stages of errors**	Physician ordering	36 (19.9)	27 (26.2)	63 (22.2)
	Transcribing	27 (14.9)	21 (20.4)	48 (16.9)
	Dispensing pharmacist	31 (17.1)	19 (18.5)	50 (17.6)
	Nurse administering	32 (17.7)	14 (13.6)	46 (16.2)
	Patient monitoring	47 (25.9)	13 (12.6)	60 (21.1)
	Others*	8 (4.4)	9 (8.7)	17 (5.9)
**Causes of errors**	Lack of knowledge about the patients†	32 (17.7)	16 (15.5)	48 (16.9)
	Lack of information about antibiotics‡	58 (32.0)	33 (32.0)	91 (32.0)
	Non-adherence to policies and procedures§	67 (37.0)	42 (40.8)	109 (38.4)
	Miscellaneous||	24 (13.3)	12 (11.7)	36 (12.7)

*Medication errors due to patient non-adherence;

^†^ information about allergy, lab tests results, concomitant medications and conditions either not available or noted;

^‡^ indication for antibiotic use, compatibility, available dosage form, dosing guidelines and route of administration;

^§^ use of abbreviation in medication ordering, incomplete medication order processed, deviation from treatment protocols, delay in dispensing, use of non-standard dosing schedule, and drug preparation errors;

^||^ illegible handwriting of physicians, memory lapse, and unavailability of drugs.

### Causality assessment

121 (59.9%) ADEs were detected among adult patients (> 18 years of age) and 81 (40.1%) among children (≤ 18 years of age). Most of the ADRs were “probable” (adults = 35.5%, children = 34.6%) and “possible” (adults = 31.4%, children = 35.8%) (Table 7).

**Table 7:** Causality assessment with respect to antibiotics class.

Antibiotics Class	ATC code	Patients > 18 years of age					Patients ≤ 18 years of age				
		Naranjo score				Total ADRs	Naranjo score				Total ADRs
		Definite* N (%)	Probable^†^ N (%)	Possible^‡^ N (%)	Doubtful^§^ N (%)		Definite* N (%)	Probable^†^ N (%)	Possible^‡^ N (%)	Doubtful^§^ N (%)	
Penicillins	J01C	1 (5.9)	10 (58.8)	2 (11.8)	4 (23.5)	**17**	0 (0.0)	5 (45.5)	2 (18.2)	4 (36.4)	**11**
Macrolides	J01FA	1 (7.7)	3 (23.1)	6 (46.2)	3 (23.1)	**13**	1 (7.7)	2 (15.4)	7 (53.8)	3 (23.1)	**13**
Cephalosporins	J01D	3 (15.8)	7 (36.8)	4 (21.1)	5 (26.3)	**19**	0 (0.0)	1 (10.0)	6 (60.0)	3 (30.0)	**10**
Fluoroquinolones	J01M	2 (11.1)	8 (44.4)	5 (27.8)	3 (16.7)	**18**	2 (22.2)	3 (33.3)	4 (44.4)	0 (0.0)	**9**
Aminoglycosides	J01G	1 (10.0)	4 (40.0)	3 (30.0)	2 (20.0)	**10**	1 (20.0)	1 (20.0)	2 (40.0)	1 (20.0)	**5**
Tetracyclines	J01AA	2 (22.2)	0 (0.0)	4 (44.4)	3 (33.3)	**9**	0 (0.0)	3 (50.0)	1 (16.7)	2 (33.3)	**6**
Lincosamide	J01FF	1 (14.3)	3 (42.9)	1 (14.3)	2 (28.6)	**7**	0 (0.0)	2 (50.0)	1 (25.0)	1 (25.0)	**4**
Carbapenem	J01DH	1 (11.1)	3 (33.3)	1 (11.1)	4 (44.4)	**9**	0 (0.0)	3 (60.0)	2 (40.0)	0 (0.0)	**5**
Glycopeptide	J01XA	0 (0.0)	2 (25.0)	5 (62.5)	1 (12.5)	**8**	2 (25.0)	3 (37.5)	1 (12.5)	2 (25.0)	**8**
Oxazolidones	J01XX	0 (0.0)	1 (16.7)	4 (66.7)	1 (16.7)	**6**	0 (0.0)	2 (66.7)	1 (33.3)	0 (0.0)	**3**
Imidazole derivatives	G01AF	0 (0.0)	2 (40.0)	3 (60.0)	0 (0.0)	**5**	1 (14.3)	3 (42.9)	2 (28.6)	1 (14.3)	**7**
**Total**		12 (9.9)	43 (35.5)	38 (31.4)	28 (23.1)	**121**	7 (8.6)	28 (34.6)	29 (35.8)	17 (20.9)	**81**

*Definite (≥9 score) ADRs are (1) followed a chronological sequence after the administration of drug or in which the drug had achieved a toxic concentration in the tissues or physiological fluid, and (3) could show improvement when the drug was withdrawal but reappeared on exposure;

^†^Probable (5–8 score) ADRs are (1) followed a chronological sequence after the administration of drug, (2) were in accordance to a recognized pattern of reactions, (3) were not confirmed by the exposure to the suspected drug but by the withdrawal of that drug, and (4) could not be described by features of the patient’s disease;

^‡^Possible (1–4) ADRs are (1) could be described by features of the patient’s disease, (2) followed a chronological sequence after the administration of drug, and (3) were in accordance to a recognized pattern of reactions;

^§^Doubtful (≤0) are factors other than a drug are associated with the reactions.

### Difference in ADEs among respondents

Logistic regression analysis examined the association between ADEs and the independent variables. Results of this analysis revealed that females had 95.3% less ADEs (OR = 0.047, 95%CI = 0.018–0.121, *p-value* = <0.001) as compared to males. Among the age groups, patients aged >18 years (OR = 0.041, 95%CI = 0.013–0.130, *p-value* = <0.001) were likely to have less ADEs as compared to patients aged ≤18 years. While examining the association between co-morbidities and ADEs, asthmatic patients (OR = 0.808, 95%CI = 0.598–1.093, *p-value* = 0.167), tuberculosis patients (OR = 0.304, 95%CI = 0.186–0.497, *p-value* = <0.001) and cystic fibrosis patients (OR = 0.527, 95%CI = 0.334–0.829, *p-value* = 0.006) were likely to have less ADEs as compared to diabetic patients. According to diagnosis, patients with acute respiratory tract infections had 99.6% less ADEs (OR = 0.004, 95%CI = 0.01−0.019, *p-value* = <0.001) and patients with skin and soft tissue infections had 95.1% less ADEs (OR = 0.49, 95%CI = 0.018−0.133, *p-value* = <0.001) as compared to the patients having urinary tract infections. Among the number of antibiotics prescribed per prescription, 2 antibiotics prescribed per prescription had 54.5% less ADEs (OR = 0.455, 95%CI = 0.319–0.650, *p-value* = <0.001) while 3 antibiotics prescribed per prescription had 1.529 times more ADEs (OR = 1.529, 95%CI = 1.063–2.198, *p-value* = 0.022) as compared to those which had 1 antibiotic prescribed per prescription (Table 8).

**Table 8:** Logistic regression analysis of factors associated with ADEs.

Characteristics		ADEs		*OR*	*95% CI*	*P-value*
		Yes	No			
Gender	Male	293 (23.5)	423 (33.9)	1.0	------	------
	Female	193 (15.5)	340 (27.2)	0.047	0.018–0.121	**<0.001**
Age	Children (≤18 years)	184 (14.7)	200 (16.0)	1.0	------	------
	Adults (>18 years)	302 (24.2)	563 (45.1)	0.041	0.013–0.130	**<0.001**
Co-morbidities	Diabetes	210 (16.8)	316 (25.3)	1.0	------	------
	Asthma	169 (13.5)	255 (20.4)	0.808	0.598–1.093	0.167
	Tuberculosis	37 (3.0)	100 (8.0)	0.304	0.186–0.497	**<0.001**
	Cystic fibrosis	70 (5.6)	92 (7.4)	0.527	0.334–0.829	**0.006**
Antibiotic indications	Urinary tract infections	198 (15.9)	264 (21.1)	1.0	------	------
	Acute respiratory tract infections	157 (12.6)	205 (16.4)	0.004	0.001–0.019	**<0.001**
	Skin and soft tissue infections	131 (10.5)	294 (23.5))	0.049	0.018–0.133	**<0.001**
Number of antibiotics prescribed per prescription	1	101 (8.1)	128 (10.2)	1.0	------	------
	2	153 (12.2)	450 (36.0)	0.455	0.319–0.650	**<0.001**
	3	232 (18.6)	185 (14.8)	1.529	1.063–2.198	**0.022**

OR = Odd Ratio, CI = Confidence Interval

## Discussion

The current study set out to determine the causality and preventability assessment of antibiotic associated ADEs among hospitalized patients. Findings showed that overall antibiotics associated ADEs were detected in 38.9% of the patients. The definitely preventable ADEs were more commonly found as compared to ADRs. Wrong drug selection was the most commonly found medication error responsible for preventable ADEs. Non-availability of national guidelines and national formularies for pediatric and geriatric population might be the possible causes of these errors [47]. Similar to our results, a study conducted in an Australian tertiary care hospital revealed that inappropriate antibiotic prescribing was the cause of antibiotic associated non-preventable ADEs in 29% of the total reported cases [48]. A study conducted in a tertiary care hospital of Kerala, India, showed that 44.9% of the total ADEs were non-preventable and cephalosporins associated ADRs were responsible for 34.7% of the total cases which affected mostly GIT and skin. Among them 10.2% of the cases were “definite” while 18.4% of the cases were “probable” and “possible” [49]. The high rate of preventable ADEs is due to the facts that clinical pharmacists in Pakistan are not actively participating in ward rounds or prescription evaluations and prescribers do not consider it necessary to report the medication errors [50]. In this study, none of the selected settings had well established pharmacovigilance center. Majority of local masses in Pakistan visit public hospitals but the ADEs reporting system in these healthcare settings is poor. This might be because of the unavailability of ADEs reporting form (e.g., yellow card scheme), low budget allocation by the government for health sector, and high patient load [51].

The most effected organs by both preventable ADEs and ADRs were GIT, kidneys and skin. Similarly, an Indian study revealed GIT as the most effected organ system due to antibiotic associated ADRs [52]. The data from Brazil had revealed that most of the ADRs affected skin (34.5%) and GIT (14.2%), and were found more common in adults (75.8%) as compared to children (7.4%) [53]. Similar to the findings of current study, a retrospective study conducted in pulmonology units of two healthcare settings of Italy had also concluded that 44.9% of the ADRs were due to antibiotics and the most affected organ was GIT [54]. This might be due to the fact that when antibiotics are administered through oral route they suppress the normal flora found in gut and can cause GIT colonization either by pathogenic or non-pathogenic organisms [55,56]. Launching national pharmacovigilance program and a central drugs standard control body under Drug Regulatory Authority of Pakistan (DRAP) can be beneficial for improving the current situation. The prime focus of this program must be the proper analysis of ADEs among inpatient and outpatient departments of both public and private healthcare settings of Pakistan. Both, federal and provincial governments must make it compulsory for healthcare professionals to report ADEs in the pharmacovigilance centers. The reports from the regional and zonal pharmacovigilance centers will be helpful in making statistical analysis of the ADEs and send these statistics to the WHO Uppsala Monitoring Centre (UMC) in Sweden.

Most of the ADEs were caused by the fluoroquinolones, imidazole derivatives and macrolides. Moreover, most of the non-preventable ADEs or ADRs were “probable” and observed in adult patients as compared to children. This is due to the reason that co-morbidities lead to poly-pharmacy, so an ADR could not be attributed to a single drug [57]. This is similar to the study conducted in a tertiary care hospital of North India where causality assessment by Naranjo score had revealed the “probable” ADRs in most of the cases [52]. A prospective study among pediatrics also reported that most of the antibiotics associated ADRs were “probable” and “possible” [58]. Antibiotics are among the most frequently prescribed medicines, and thus may cause the incidence of ADRs to occur at higher rate. Similarly, in a previously published study, fewer ADRs were detected in such healthcare settings where the trend of prescribing antibiotics is infrequent [59]. This is the reason that the guidelines of Centers for Disease Control and Prevention (CDC) does not recommend the physicians to prescribe unnecessary antibiotics especially in hospitalized patients [60]. Moreover the identification of causal association of antibiotics with ADRs helps to prevent iatrogenic complication, therapy optimization and establishment of barriers to prevent the chances of recurrence [61].

Logistic regression analysis showed statistically significant differences among different genders, age groups, co-morbidities, diagnosis and number of antibiotics prescribed per prescription of patients in the study. Less ADEs were found in patients having female gender, >18 years of age, suffering from tuberculosis and acute respiratory tract infections and who were prescribed 2 antibiotics per prescription. Similar to our findings a prospective cross-sectional study revealed directly proportional relation between that ADEs and factors like age and poly-pharmacy [62]. There are various physiological and pharmacological variations among children and adults that cause the therapeutic agents to respond differently among these two age groups [63]. A retrospective study conducted on Chinese pediatric inpatients also showed that occurrence of ADEs was significantly associated with number of drugs but not with other factors like age and gender [64]. This is primarily because of the reason that the risk of drug interactions increases when more number of drugs are prescribed to the patients which leads to the development of ADEs [65]. Similar to findings of previously published studies [66–68], a significant association was found in current study between male gender and ADEs. In contrast to this, other studies depict that antibiotics associated ADEs are more commonly found among females as compared to males [69,70]. While, some studies showed no significant association of ADEs with gender [64,71,72]. This is merely because of the fact that gender differences may not only include biologic differences but other factors like social, cultural, behavioral and physiological dissimilarities have an impact on it [73]. Furthermore, co-morbidities are also attributed as a significant factor for developing ADEs. Findings also suggest that antibiotics associated ADEs were more commonly found among those patients who had diabetes mellitus (DM) as co-morbidity. This might be because of the fact that DM can impair renal functions and negatively effects the metabolism of drugs which makes the patients more prone towards the development of ADEs [74].

This study has some limitations. First, the findings of present study cannot be generalized to entire country; however, since the condition of healthcare sector and pharmacovigilance is similar across the country so it is likely that results are similar for other tertiary care hospitals as well. Second, since it was a cross-sectional study so long term effects of ADEs could not be traced. Future longitudinal studies may address these aspects. The outcomes of treatment interventions like rechallenge and dechallenge were not measured in this study, therefore very few cases were categorized as definite. Also, the Hawthorne effect could have affected the result because physicians, nurses and other paramedical staff were well aware of the study.

## Conclusion

The present study concluded that the most commonly prescribed antibiotics among hospitalized patients were fluoroquinolones, macrolides and imidazole derivatives. The antibiotic associated ADEs were common in pediatric patients as compared to adults. The preventability assessment showed that most of the ADEs observed among hospitalized patients were preventable and caused by MEs such as wrong drug and wrong dose errors during the stage of physician ordering and patient monitoring. According to causality assessment most of the non-preventable ADEs or ADRs among adults were probable and among children were possible. The most affected organ system by antibiotics associated ADEs among all age groups was gastrointestinal tract. Furthermore, logistic regression analysis revealed that less ADEs were found in patients having female gender, >18 years of age, tuberculosis disease, acute respiratory tract infections and 2 antibiotics prescribed per prescription. The findings of this study might make the healthcare policy makers aware about the current situation regarding pharmacovigilance system who may take adequate steps for formulating appropriate strategies to prevent the patients from untoward effects of improper use of antibiotics.

## Supporting information

S1 Appendix Characteristics of selected hospitals. (DOCX)

S2 Appendix Questionnaire of the study. (DOCX)

S3 Appendix Definitions of different terms. (DOCX)

S1 File SPSS file. (SAV)

## Abbreviations

ADRs: Adverse drug reactions
DRAP: Drug Regulatory Authority of Pakistan
UMC: Uppsala Monitoring Centre
WHO: World Health Organization
