# Supplementary material for: Causality and preventability assessment of adverse drug reactions and adverse drug events of antibiotics among hospitalized patients: A multicenter, cross-sectional study in Lahore, Pakistan
Source: PLoS One. 2018 Jun 27;13(6):e0199456. doi: 10.1371/journal.pone.0199456 (PMC6021047; doi:10.1371/journal.pone.0199456)
Supplement: S1 Appendix — (DOCX) [file pone.0199456.s001.docx]

**Characteristics of selected hospitals**

| **Sr. no.** | **Characteristics** | **Mayo hospital** | **Jinnah hospital** | **General hospital** | **Services hospital** |
| --- | --- | --- | --- | --- | --- |
| 1 | Number of beds | 2,400 | 1, 500 | 1, 300 | 1, 196 |
| 2 | Inpatients visit last year | 343, 114 | 217, 245 | 134, 491 | 125, 868 |
| 3 | Prescribers/Medical officers | 550 | 348 | 300 | 274 |
| 4 | Nurses | 500 | 313 | 271 | 249 |
| 5 | Pharmacists/Dispensers | 30 | 19 | 16 | 14 |
| 8 | ^*^Other paramedical staff | 671 | 445 | 382 | 304 |
| 10 | Existence of pharmacovigilance center in hospital | No | No | No | No |
| 11 | Maintenance of ADR registers | No | No | No | No |

^*^Other Paramedical staff includes; medical technicians, ward boys, and sweepers
