# Supplementary material for: Causality and preventability assessment of adverse drug reactions and adverse drug events of antibiotics among hospitalized patients: A multicenter, cross-sectional study in Lahore, Pakistan
Source: PLoS One. 2018 Jun 27;13(6):e0199456. doi: 10.1371/journal.pone.0199456 (PMC6021047; doi:10.1371/journal.pone.0199456)
Supplement: S2 Appendix — (DOCX) [file pone.0199456.s002.docx]

**Questionnaire of the study**

**Section 1: General information**

Name of patient Age (years)

Date of hospitalization Weight (Kg)

Height of patient (cm) Gender

Hospital and ward

Name of interviewer

Name of attending physician

Name of attending nurse

Name of clinical pharmacist

Name of dispensing pharmacist

**Section 2: Drug and Disease history**

What are the drugs (especially antibiotics) from which the patient was allergic?

What are the co-morbidities associated with the patients?

What medications are administered to patients prior to the initiation of antibiotics?

**Section 3: Patient’s medication information**

What antibiotics are prescribed on the basis of differential diagnosis?

Dose Dosage form Route Frequency

Antibiotics belong to which class?

Penicillins Macrolides Cephalosporins

Fluoroquinolones Aminoglycosides Tetracyclines

Lincosamide Carbapenem Glycopeptide

Oxazolidones Imidazole derivatives

Is there any drug interaction found?

Yes No

**Section 4: Sign and symptoms appear after initiation of antibiotics**

| **Organ system/organs** | **Description of signs and symptoms** |
| --- | --- |
| Cardiovascular system (CVS) |  |
| Gastrointestinal system (GIT) |  |
| Hepatobiliary system |  |
| Respiratory system |  |
| Skin |  |
| Central nervous system (CNS) |  |
| Kidneys |  |
| Ear, Nose, throat (ENT) |  |

**Section 5: Patient’s lab report**

Electrocardiogram (ECG)

Total leucocyte count (TLC) (cells/µL)

Erythrocytes count (cells/µL)

Hemoglobin (g/dL)

Platelet count (cells/µL)

Total bilirubin (mg/dL)

Aspartate aminotransferase (AST) (units/L)

Alanine transaminase (ALT) (units/L)

Alkaline phosphatase (ALP) test (units/L)

Serum creatinine (mg/dL)

Immunoglobulins (mg/dL)

Others

**Were adverse drug events (ADEs) occur?**

**Yes No**

**Section 6: Preventability assessment**

| **Sr. no.** | **Schmuck and Thornton Criteria** | **Yes** | **No** |
| --- | --- | --- | --- |
| **Definitely preventable ADEs** | | | |
| 1. | Was there a history of allergy or previous reaction to the drug? |  |  |
| 2. | Was the drug involved inappropriate for the patient’s clinical condition? |  |  |
| 3. | Was the dose, route, or frequency of administration inappropriate for patient’s age, weight or disease state? |  |  |
| 4. | Was toxic serum drug concentration or lab monitoring test documented? |  |  |
| 5. | Was there a known treatment for ADEs? |  |  |
| **Probably preventable ADEs** | | | |
| 6. | Was therapeutic drug monitoring or other necessary lab test not performed? |  |  |
| 7. | Was the drug interaction involved in ADEs? |  |  |
| 8. | Was poor compliance involved in ADE? |  |  |
| 9. | Were preventative measures not prescribed or administered to the patient? |  |  |
| **Non-preventable ADEs or ADRs** | | | |
| 10. | If all the above criteria not fulfilled. |  |  |

On the basis of Schmuck and Thornton criteria, ADEs are,

Definitely preventable ADEs probably preventable ADEs

Non-preventable ADEs / adverse drug reaction (ADRs)

**Section 7: Medication errors**

***i) Patient information***

Date of report

Name of reporter

Patient location at the time of error

Date and time of error

***ii) Medication order information***

How was order written?

Did the patient receive the medication?

Yes, (if yes then what did patient receive [dose, route and time of administration]?)

No, (if no then how was error intercepted?

***iii) Categorization of medication errors***

*A.Prescribing/ordering* *(check all that apply)*

Order written on wrong patient

Order written for wrong drug

Drug was inappropriate for indication

Patient was allergic to drug

Drug-drug or drug-disease interactions

Order written for wrong or dose was not adjusted

Order written for wrong dosing schedule

Order written for wrong route

*B.Transcription/verification (check all that apply)*

Order transcribed on wrong patient

Order transcribed for wrong drug

Order transcribed for wrong dose

Order transcribed for wrong dosing schedule

Order transcribed for wrong route

*C.Dispensing (check all that apply)*

Medication dispensed to wrong patient

Wrong medication dispensed

Wrong dose dispensed

Medication dispensed at wrong time

Wrong dosage form dispensed

*D.Administration (check all that apply)*

Medication administered to wrong patient

Wrong medication administered to patient

Wrong dose administered

Medication administered at wrong time

Medication administered via wrong route

*E.Monitoring*

Necessary monitoring not ordered

Necessary monitoring not performed

Monitoring results not noted

***iv) Possible causes of medication errors***

A.Patient knowledge deficiency

Allergy information not available or noted

Concomitant medications not available or noted

Concomitant conditions not available or noted

Clinical information or lab values not available or noted

B.Medication knowledge deficiency

Indications for the use of medications

Available dosage form

Guidelines related to dosing

Appropriate route for administration

Drug compatibility

C.Non-adherence to policy and procedures

Use of abbreviation in medication ordering

Incomplete medication order processed

Deviation from treatment protocols

Delay in dispensing

Use of non-standard dosing schedule

Drug preparation error

D.Miscellaneous

Illegible handwriting of physicians

Memory lapse

Unavailability of drugs

**Section 8: Naranjo algorithm scale**

| **Sr. no.** | **Questions** | **Yes** | **No** | **Don’t know/Not performed** | **Score** |
| --- | --- | --- | --- | --- | --- |
| 1. | Are there previous *conclusive* reports on this reaction? | +1 | 0 | 0 |  |
| 2. | Did the adverse event occur after the suspected drug was administered? | +2 | -1 | 0 |  |
| 3. | Did the adverse reaction improve when the drug was discontinued or a *specific* antagonist was administered? | +1 | 0 | 0 |  |
| 4. | Did the adverse reaction reappear when the drug was readministered? | +2 | -1 | 0 |  |
| 5. | Are there alternative causes (other than the drug) that could have on their own caused the reaction? | -2 | +1 | 0 |  |
| 6. | Did the reaction reappear when a placebo was given? | -1 | +1 | 0 |  |
| 7. | Was the blood detected in the blood (or other fluids) in concentrations known to be toxic? | +1 | 0 | 0 |  |
| 8. | Was the reaction more severe when the dose was increased or less severe when the dose was decreased? | +1 | 0 | 0 |  |
| 9. | Did the patient have a similar reaction to the same or similar drugs in *any* previous exposure? | +1 | 0 | 0 |  |
| 10. | Was the adverse event confirmed by any objective evidence? | +1 | 0 | 0 |  |
| **Total score** | | | | |  |

On the basis of total score, ADR is;

Definite (score >9) Probable (score 5-8)

Possible (score 1-4) Doubtful (score 0)
