# Supplementary material for: Causality and preventability assessment of adverse drug reactions and adverse drug events of antibiotics among hospitalized patients: A multicenter, cross-sectional study in Lahore, Pakistan
Source: PLoS One. 2018 Jun 27;13(6):e0199456. doi: 10.1371/journal.pone.0199456 (PMC6021047; doi:10.1371/journal.pone.0199456)
Supplement: S3 Appendix — (DOCX) [file pone.0199456.s003.docx]

**Definitions of different terms**

| **Terms** | **Definitions** |
| --- | --- |
| Inpatients | Those who acquired bed in hospital and admitted for >24 hours. |
| Child | A patient whose age is ≤18 years of age. |
| Adult | A patient who is >18 years of age. |
| Wrong drug | Occur when the drug is inappropriate to the patient’s medical condition. |
| Wrong dose | Prescribed dose was ±10% of the recommended dose in the guidelines. |
| Wrong route | Prescribed drug was administered other than the recommended route by the guidelines. |
| Wrong time | Occur when the medication is administered before or after the scheduled administration time (if there is >1 hour difference between the scheduled time and administration time). |
| Deteriorated drug | Administration of drug after its expiry date or for which the physical or chemical dosage-form integrity has been compromised. |
| Omission | Occur when the ordered dose was failed to administer to the patient before the next schedule. |
| Wrong dosage form error | Administration of dosage form other than the prescribed dosage form. |
| Non-adherence | Occur when patient don’t show compliance with the prescribed medication. |
| Monitoring error | Occur when therapeutic drug monitoring and other required lab test not performed. |
